# Review of "Systems Biology in Practice" by Edda Klipp, Ralf Hertwig, Axel Kowald, Christoph Wierling and Hans Lehrach

**DOI:** 10.1186/1742-4682-2-34

**Published:** 2005-08-26

**Authors:** Paul S Agutter

**Affiliations:** 1 Theoretical and Cell Biology Consultancy, 26 Castle Hill, Glossop, Derbyshire, UK

Systems biology – the study of such functional networks as cellular metabolism, signalling and gene expression – is a rapid growth area, as illustrated by many recent publications in *Theoretical Biology and Medical Modelling *and other journals. Its emergence has been fostered by the development of automated, high-throughput techniques such as DNA microarrays, of the ever-increasing storage capacity and calculating speed of computers, of new mathematical tools, and of modern electronic communications. Its impact on biological research is already evident: traditional data (e.g. the results of single-gene studies) are being reinterpreted in a broader cellular context, radically new research strategies are appearing, novel insights into the integration of cell systems are being gained, and the prospects for advances in medicine, biotechnology, ecology and other areas of applied science and technology seem, to the optimistic, almost limitless. To write a book surveying the concepts, methods and potential applications of this nascent though already wide field was a challenging enterprise, but the authors of *Systems Biology in Practice *have largely achieved their objective. This is a timely volume that should be welcomed both by practising systems biologists and by newcomers to the field.

The book is divided into three parts. Part I (chapters 1–4), occupying about a quarter of the main text, introduces the relevant biological and mathematical concepts and experimental techniques. Part II (chapters 5–12), occupying the following two-thirds of the book, focuses on the three main facets of systems biology as it stands today (metabolism, signal transduction and gene expression), but it also includes brief surveys of the cell cycle and ageing, an interesting chapter on evolution and self-organization, a discussion of data integration methods, and a brief but lively speculation about future directions and applications. The short final part (chapters 13–14) is a descriptive list of currently-available internet databases and tools and modelling tools, assessing the advantages and disadvantages of each. Every concept and method introduced throughout the book is illustrated by at least one boxed example – a well-attested and effective pedagogical device – and most of the examples used are clear and appropriate. The overall organization of the text is lucid, the index is thorough, and the publishers are to be commended on a very well-produced volume.

Inevitably, since this is a pioneering work on the subject, there are flaws. Informed readers will notice omissions; for example, the otherwise excellent account of metabolic control theory in chapter 5 makes no mention of the biochemical systems theory of Savageau, Voit and their colleagues, which some authorities consider conceptually and methodologically superior in certain applications. The balance between sections of the text seems odd in places. In chapter 3, for instance, after clear and well-directed introductions to linear algebra and ordinary differential equations, the authors launch into an account of advanced statistical theory (section 3.4.1) that has little direct relevance to the remainder of the book – in contrast to the remainder of section 3.4, where various statistical techniques and their practical applications are well described – and would be better placed in an appendix, if not omitted. Chapter 5 begins with 20 pages of elementary thermodynamics and enzymology, surely elementary for most readers, and ends with the same amount of text – a mere 20 pages – on metabolic control theory, which is less likely to be familiar and is far more mathematically sophisticated. Much of the account of ageing in chapter 7 is devoted to the defective mitochondria theory – reasonably, in that one of the authors (Kowald) has been a major contributor to this theory, but less reasonably in that several alternative models of ageing in the literature are scarcely considered. There are several misprints: for example, equation 3.21 (p. 63) is incorrect, 'sites' is written for 'sides' (p. 67) and 'mitochondrium' for 'mitochondrion' (p. 168). In places, the writing would have benefited from the assistance of a native English speaker; odd phrasing such as 'negative definite' for 'always negative' (p. 74, example 3–10 and surrounding text) is distracting. Perhaps more importantly for some readers, the authors have concentrated throughout on ordinary differential equation models. Stochastic modelling is mentioned where it is potentially relevant, but only briefly, and this might be the most serious imbalance in the book; as the authors admit, the continuum hypothesis is unlikely to be valid in modelling many aspects of signal processing and transcriptional regulation.

Because of the rate of progress in systems biology, it seems likely that a second edition of this book will appear before many years have passed. It is to be hoped that these (mostly minor) defects will be eliminated in the next edition. However, they detract little from the value of the work as it stands. I expect to consult it regularly over the foreseeable future, and I am confident that biologists everywhere will benefit from having a copy to hand.

